# High-fat diet-induced gut microbiota functional reprogramming is associated with metabolic inflammation and cardiac remodeling through the gut-heart axis in mice

**DOI:** 10.3389/fimmu.2026.1912486

**Published:** 2026-08-25

**Authors:** Yan Feng, Kai Yang, Zhuan Qu, Tengyan Liu, Dan Yang, Su Wei, Yaxin He, Xiaotian Wang, Yun Bai, Fangyuan Wang, Yu Zhao, Lifen Dai

**Affiliations:** 1 Department of Internal Medicine, Fuwai Yunnan Cardiovascular Hospital, Kunming, China; 2 Department of Endocrinology, People's Hospital of Shuangjiang Autonomous County, Lincang, Yunnan, China; 3 Yunnan Key Laboratory of Innovative Diagnosis and Treatment of Circulatory Diseases, Fuwai Yunnan Hospital, Chinese Academy of Medical Sciences, Affiliated Cardiovascular Hospital of Kunming Medical University, Kunming, China

**Keywords:** cardiac remodeling, gut microbiota, gut-heart axis, high-fat diet, metabolic syndrome

## Abstract

**Background:**

Gut microbiota alterations have been implicated in the progression of metabolic syndrome (MetS), which increases the risk of cardiovascular disease (CVD) and is influenced by lifestyle factors. However, the specific gut microbiota changes and their functional roles in MetS remain incompletely understood.

**Aims:**

We aimed to identify key gut microbial taxa associated with MetS and to explore their potential functional implications.

**Methods:**

Six-week-old male C57BL/6J mice were fed a high-fat diet (HFD) or normal chow diet (NCD) for 16 weeks. Body weight and adiposity, metabolic parameters (fasting blood glucose, oral glucose tolerance test, and serum insulin), systemic inflammatory markers (IL-6, TNF-α, CRP, and high-sensitivity CRP), and cardiovascular phenotypes (total cholesterol, triglycerides, low-density lipoprotein cholesterol, and high-density lipoprotein cholesterol) were assessed. Cardiac remodeling was evaluated by heart weight-to-body weight ratio and myocardial histopathology using hematoxylin and eosin (H&E) and Masson’s trichrome staining. Cecal contents were collected for 16S rRNA gene sequencing.

**Results:**

HFD feeding induced a MetS-like phenotype characterized by obesity, insulin resistance, dyslipidemia, systemic inflammation, and early cardiac remodeling in mice. 16S rRNA sequencing revealed significant alterations in gut microbial composition, including enrichment of inflammation-associated taxa such as *Escherichia-Shigella*, *Parasutterella*, *Faecalibaculum*, and the *Clostridium innocuum group*, together with depletion of potentially beneficial bacteria including *Roseburia*, *Alistipes*, *Parabacteroides*, *Prevotellaceae_UCG-001*, and *Rikenellaceae_RC9_gut_group*. Functional prediction analyses indicated alterations in microbial pathways related to phosphotransferase systems, xenobiotic degradation, infectious disease signaling, DNA repair, transcriptional regulation, replication, recombination, and mobile genetic elements. BugBase phenotype analysis further demonstrated increased facultatively anaerobic, stress-tolerant, biofilm-forming, and potentially pathogenic microbial phenotypes following HFD exposure.

**Conclusion:**

Our findings suggest that HFD-induced Mets is associated with gut microbiota dysbiosis and functional remodeling, suggesting a potential involvement of microbiota-mediated metabolic pathways and host-microbiota interactions in cardiometabolic abnormalities.

## 1 Introduction

Obesity is a well-established cardiometabolic risk factor with a high prevalence among both children and adults, representing a major global public health challenge (1). As a primary driver of metabolic syndrome (MetS), obesity substantially increases the likelihood of developing this multifactorial disorder (2, 3). MetS is characterized by a cluster of metabolic abnormalities, including insulin resistance or overt diabetes mellitus, atherogenic dyslipidemia, central obesity, hypertension, chronic low-grade inflammation, and an increased risk of cardiovascular disease (CVD) (4, 5). Multiple risk factors contribute to the development of MetS, including obesity, insulin resistance, physical inactivity, vitamin D deficiency, sleep disturbances, hypercortisolism, systemic and tissue inflammation, gut microbiota alterations, and genetic predisposition (6, 7). Growing evidence suggests that lifestyle modification is one of the most effective strategies for preventing and managing MetS (8, 9).

MetS not only impairs metabolic homeostasis but also exerts profound adverse effects on the cardiovascular system (10). The components of MetS promote a chronic low-grade systemic inflammatory state, which is associated with increased sympathetic nervous system activity, cardiac hypertrophy, arrhythmias, endothelial dysfunction, and atherosclerosis (11–14). Consequently, MetS imposes a substantial burden on cardiovascular health, as demonstrated by both clinical and experimental studies. Individuals with MetS have been reported to exhibit a 70% higher risk of sudden cardiac death (SCD), even in the absence of a prior history of coronary heart disease (CHD) (15). Despite extensive research, the mechanisms underlying MetS-associated cardiovascular injury remain incompletely understood.

The gut microbiota, often referred to as a “virtual organ,” comprises trillions of microorganisms residing within the gastrointestinal tract (GIT). These microorganisms play essential roles in maintaining host health by supporting intestinal barrier integrity, modulating immune responses, preventing pathogen colonization, and regulating various metabolic processes (16, 17). Diet and lifestyle are considered the major determinants of gut microbial composition and function (18). Accumulating evidence indicates that a high-fat diet (HFD) can induce profound alterations in the gut microbiota, including reduced microbial diversity, expansion of Proteobacteria, depletion of short-chain fatty acid (SCFA)-producing bacteria, increased dysbiosis, and enhanced inflammatory responses, thereby contributing to the development and progression of multiple diseases (19, 20). For example, HFD has been shown to disrupt the peripheral kynurenine pathway in a gut microbiota-dependent manner in C57BL/6 mice (21). HFD-induced gut microbial dysbiosis has also been implicated in colorectal tumorigenesis through metabolomic alterations, including elevated lysophosphatidic acid levels and impaired intestinal barrier function (22). Furthermore, HFD-mediated reductions in histidine-producing bacteria contribute to the pathogenesis of non-alcoholic fatty liver disease (NAFLD) (23), while decreased microbial diversity has been associated with HFD-induced cognitive impairment (24). In addition, HFD-induced accumulation of L-saccharopine has been reported to mediate ovarian dysfunction (25). Notably, HFD has also been shown to induce cardiometabolic disorders accompanied by gut microbiota impairment in rodent models (26). However, the specific role of gut microbiota dysbiosis in mediating HFD-induced cardiometabolic dysfunction and cardiac injury remains poorly understood.

In the present study, we established an HFD-induced mouse model to mimic the pathological features of MetS and investigate the relationship between metabolic dysfunction, cardiac injury, and gut microbiota dysbiosis. Furthermore, we aimed to identify specific gut microbial taxa associated with HFD-induced cardiometabolic abnormalities and to explore the underlying regulatory mechanisms involved in this process.

## 2 Methods

### 2.1 Animals

All animal procedures were performed in accordance with the Guide for the Care and Use of Laboratory Animals and were approved by the Animal Care and Use Committee of Fuwai Yunnan Cardiovascular Disease Hospital (Approval No. KMMUX202603646).

A total of twelve specific pathogen-free (SPF) male C57BL/6J mice (six weeks old) were purchased from Servarelife Co., Ltd. (License No. SYXK (Er) 2026-0167). Mice were housed under SPF conditions with free access to food and water and allowed to acclimate for one week prior to the experiment. Following acclimatization, mice were randomly assigned to two groups (n = 6 per group; 3 mice per cage): a normal control group (Ctrl) and a high-fat diet group (HFD). Mice in the Ctrl group were fed a standard chow diet containing 10% kcal from fat, 20% kcal from protein, and 70% kcal from carbohydrates. Mice in the HFD group received a high-fat diet containing 60% kcal from fat, 20% kcal from protein, and 20% kcal from carbohydrates (27). The dietary intervention was continued for 16 consecutive weeks. Body weight and food intake were recorded throughout the experimental period. The 16-week HFD intervention was selected based on previous studies (28–31) showing that this duration is sufficient to induce obesity, metabolic syndrome-like alterations, systemic inflammation, and early cardiac remodeling in C57BL/6J mice.

At the end of the study, mice were fasted for 12 h before sample collection. Blood samples were obtained from the orbital venous plexus. Mice were euthanized by CO_2_ inhalation using a gradual fill rate of 30-70% chamber volume/min, followed by cervical dislocation to ensure death. Hearts and epididymal adipose tissues were harvested, weighed, snap-frozen in liquid nitrogen, and stored at -80 °C for subsequent analyses.

### 2.2 Fasting blood glucose test

At the end of the experimental period, fasting blood glucose (FBG) levels were measured after a 12-h fasting period.

### 2.3 Oral glucose tolerance test and serum insulin measurement

For the oral glucose tolerance test (OGTT), mice were fasted overnight and orally administered 20% glucose solution (100 μL/10 g body weight). Blood glucose levels were measured from tail vein blood at 0, 15, 30, 60, 90, and 120 min after glucose administration using a Chemray 800 biochemical analyzer.

Serum insulin (INS) concentrations were determined using a commercially available enzyme-linked immunosorbent assay (ELISA) kit (CEA448Mu, Cloud-Clone Corp., Wuhan, China) according to the manufacturer’s instructions.

### 2.4 Assessment of the inflammatory cytokines

Serum levels of interleukin-6 (IL-6; 88-7064, Invitrogen, USA), tumor necrosis factor-α (TNF-α; 88-7324, Invitrogen, USA), C-reactive protein (CRP; EK294/2-48, Multi Sciences, China), and high-sensitivity C-reactive protein (hsCRP; SMK3108B, Meikebio, China) were measured using commercial ELISA kits according to the manufacturers’ instructions.

### 2.5 Serum biochemical analysis

Serum levels of glucose, total cholesterol (TC), triglycerides (TG), low-density lipoprotein cholesterol (LDL-C), and high-density lipoprotein cholesterol (HDL-C) were determined using commercially available assay kits (Servicebio Co., Ltd., Wuhan, China) according to the manufacturer’s protocols.

### 2.6 Histological analysis

Myocardial tissues were fixed in 4% paraformaldehyde for 24 h, embedded in paraffin, and sectioned for histological examination. Sections were stained with hematoxylin and eosin (H&E) and Masson’s trichrome staining. Histological images were captured under a light microscope (Olympus, Tokyo, Japan) at ×40 magnification.

### 2.7 Fecal sample collection and microbiome analysis

Cecal contents were collected aseptically at the end of the experiment and stored at -80 °C until analysis. Microbial genomic DNA was extracted using a Fecal DNA Isolation Kit (Servicebio, Wuhan, China) according to the manufacturer’s instructions. The V4 hypervariable region of the bacterial 16S rRNA gene was amplified using unique barcoded primers to construct individual amplicon libraries.

Amplicons (~255 bp) were sequenced on the DNBSEQ-G99RS platform (MGI Tech Co., Ltd., Shenzhen, China) using single-end sequencing. Raw sequencing reads were processed using a standardized bioinformatics pipeline. Briefly, low-quality sequences were filtered using Trimmomatic (32), followed by primer trimming using Cutadapt to generate high-quality clean reads (33). Denoising, chimera removal, and amplicon sequence variant (ASV) generation were subsequently performed using the DADA2 pipeline implemented in QIIME2 (34).

Taxonomic annotation was conducted using a 16S rRNA reference database with a confidence threshold of 0.8. ASVs with a mean relative abundance of less than 0.005% were excluded from further analyses. Taxonomic abundance profiles were summarized at multiple taxonomic levels, including phylum, class, order, family, genus, and species. Taxonomic composition was visualized using stacked bar charts generated in Microsoft Excel.

Alpha-diversity and beta-diversity analyses were performed to assess microbial diversity within and between samples, respectively. Principal coordinates analysis (PCoA) was conducted using QIIME2 to visualize differences in microbial community structure among groups, and three-dimensional PCoA plots were generated using Emperor.

Differential abundance analyses were performed using multiple statistical and machine-learning approaches to identify significantly altered microbial taxa between groups. Functional prediction analyses, including KEGG, COG, BugBase, and FAPROTAX, were subsequently conducted based on ASV abundance profiles to infer potential microbial functions. Finally, genus-level correlation analyses were performed to explore associations among microbial taxa and between microbial communities and host phenotypic parameters.

### 2.8 Statistical analysis

All data are presented as the mean ± standard deviation (SD). Statistical analyses and data visualization were performed using GraphPad Prism 9.0 (GraphPad Software, San Diego, CA, USA). Comparisons between two groups were conducted using the unpaired Student’s *t*-test for normally distributed data and the Mann-Whitney U test for non-normally distributed data. A two-tailed *P* value < 0.05 was considered statistically significant.

## 3 Results

### 3.1 High-fat diet successfully induces obesity, metabolic dysfunction, and insulin resistance in mice

**Figure 1:**
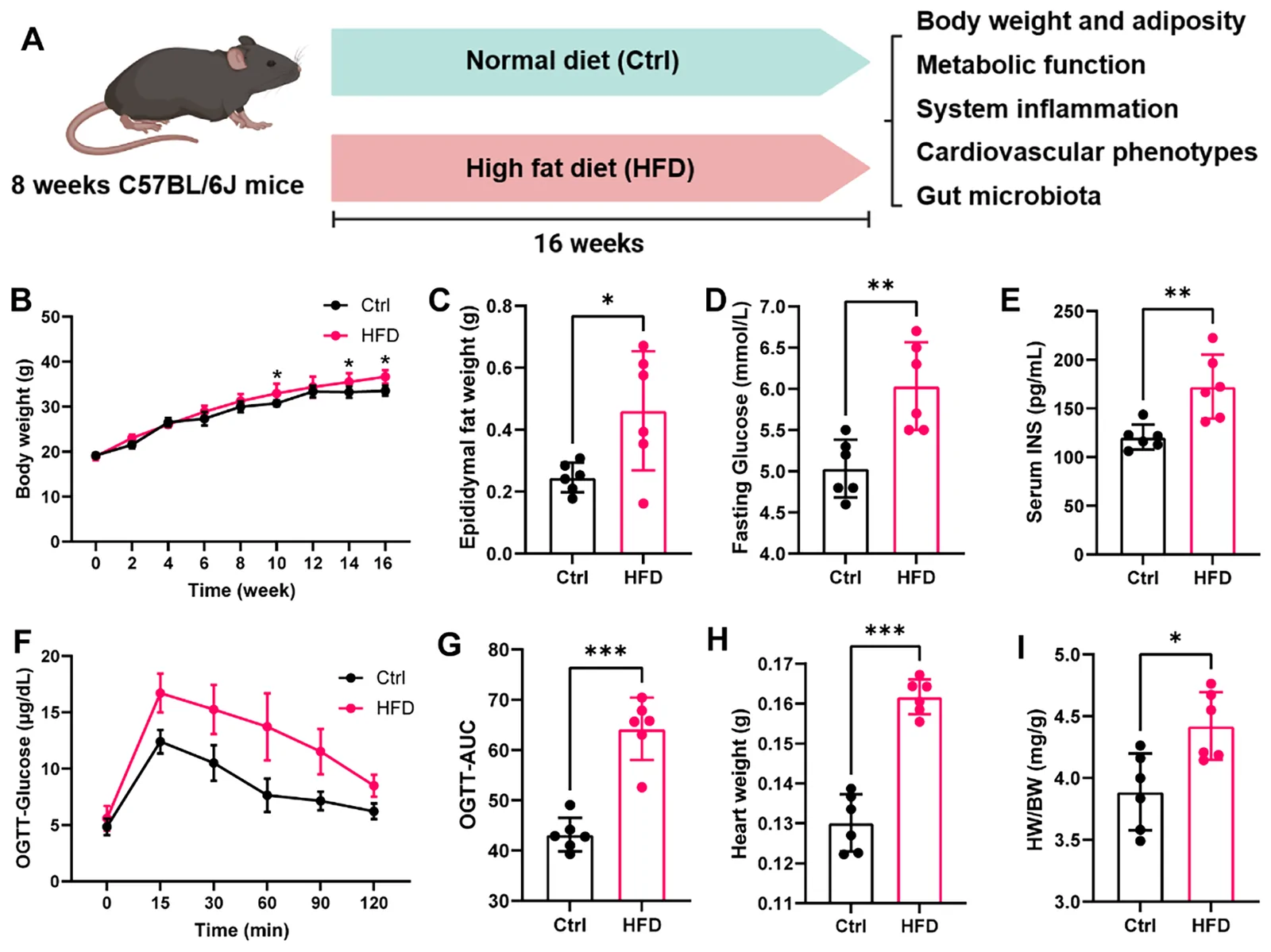
High-fat diet (HFD) successfully induces obesity, metabolic dysfunction, and insulin resistance in mice. **(A)** Schematic illustration of the experimental design and animal grouping. **(B, C)** Body weight **(B)** and epididymal fat weight **(C)** in the Ctrl and HFD groups. **(D-G)** Fasting blood glucose **(D)**, serum insulin levels **(E)**, oral glucose tolerance test (OGTT) curves **(F)**, and the corresponding area under the curve (AUC) values **(G)**. **(H-I)** Heart weight **(H)** and heart weight-to-body weight ratio (HW/BW) **(I)** in the indicated groups. Data are presented as mean ± SD (n = 6 per group). *P < 0.05, **P < 0.01, ***P < 0.001. OGTT, oral glucose tolerance test; AUC, area under the curve; HW/BW, heart weight-to-body weight ratio.

To establish a high-fat diet (HFD)-induced metabolic syndrome model, body weight, glucose metabolism, lipid profiles, inflammatory markers, and tissue morphology were systematically evaluated (**Figure 1A**).

Compared with control (Ctrl) mice, HFD-fed mice exhibited a significant increase in body weight and epididymal adipose tissue mass (**Figures 1B, C**), indicating successful induction of obesity. Fasting blood glucose and serum insulin levels were markedly elevated in HFD mice compared with Ctrl mice (**Figures 1D, E**). Consistently, oral glucose tolerance tests (OGTTs) demonstrated impaired glucose tolerance in HFD-fed mice, as evidenced by increased blood glucose levels and a significantly greater area under the curve (AUC) (**Figures 1F, G**), indicating the development of insulin resistance and metabolic dysfunction.

Furthermore, both heart weight and the heart weight-to-body weight ratio were significantly increased in HFD mice (**Figures 1H, I**), suggesting the presence of early cardiac remodeling under metabolic stress. Collectively, these findings demonstrate that long-term HFD feeding successfully induces obesity, glucose intolerance, insulin resistance, and metabolic disturbances in mice.

### 3.2 HFD induces dyslipidemia and systemic low-grade inflammation

**Figure 2:**
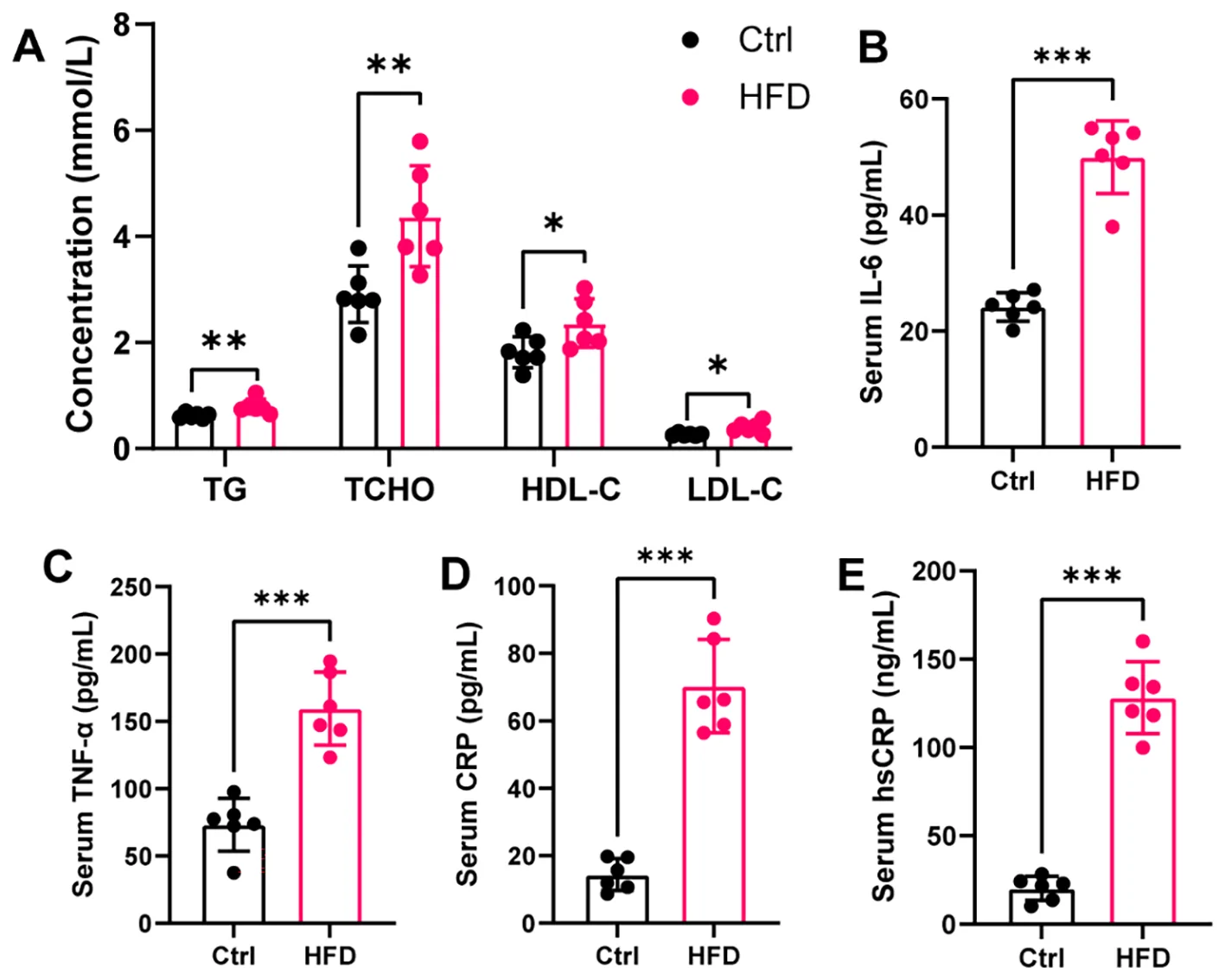
HFD induces dyslipidemia and systemic low-grade inflammation. **(A)** Serum levels of total cholesterol (TCHO), triglycerides (TG), high-density lipoprotein cholesterol (HDL-C), and low-density lipoprotein cholesterol (LDL-C) in the indicated groups. **(B-E)** Serum levels of IL-6 **(B)**, TNF-α **(C)**, CRP **(D)**, and hs-CRP **(E)** in the Ctrl and HFD groups. Data are presented as mean ± SD (n = 6 per group). *P < 0.05, **P < 0.01, ***P < 0.001. TCHO, total cholesterol; TG, triglycerides; HDL-C, high-density lipoprotein cholesterol; LDL-C, low-density lipoprotein cholesterol.

Serum biochemical analysis revealed significant alterations in lipid metabolism following HFD feeding. Compared with Ctrl mice, HFD-fed mice exhibited markedly elevated serum levels of triglycerides (TG), total cholesterol (TC), high-density lipoprotein cholesterol (HDL-C), and low-density lipoprotein cholesterol (LDL-C) (**Figure 2A**), indicating substantial disruption of lipid homeostasis.

In parallel, HFD markedly increased circulating inflammatory markers. Serum levels of interleukin-6 (IL-6) (**Figure 2B**), tumor necrosis factor-α (TNF-α) (**Figure 2C**), C-reactive protein (CRP) (**Figure 2D**), and high-sensitivity C-reactive protein (hsCRP) (**Figure 2E**) were all significantly elevated in HFD-fed mice compared with controls. These findings suggest that HFD induces chronic low-grade systemic inflammation in addition to metabolic abnormalities, resulting in a phenotype closely resembling human metabolic syndrome.

### 3.3 HFD induces myocardial inflammation and fibrotic remodeling

**Figure 3:**
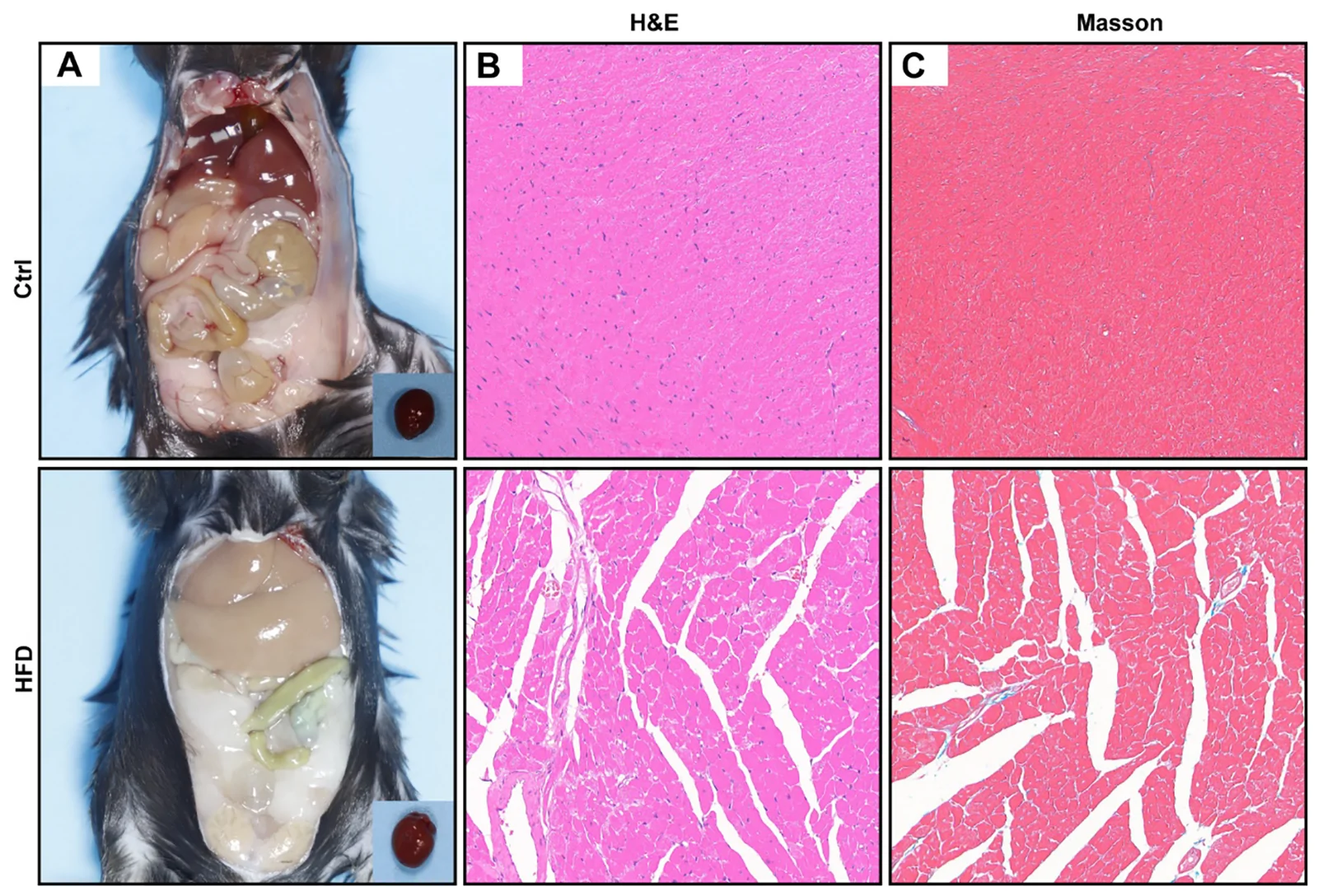
HFD induces myocardial inflammation and fibrotic remodeling. **(A)** Representative images of tissues collected from Ctrl and HFD mice. **(B, C)** Representative hematoxylin and eosin (H&E) staining **(B)** and Masson’s trichrome staining **(C)** of heart sections from the indicated groups.

Histopathological analyses were performed to evaluate tissue injury induced by HFD feeding. Although no obvious hepatic lipid accumulation was observed, significant pathological alterations were detected in cardiac tissue (**Figures 3A–C**). Hematoxylin and eosin (H&E) staining revealed mild increased inflammatory cell infiltration and disorganized myocardial architecture in HFD-fed mice compared with Ctrl mice (**Figure 3B**). Moreover, Masson’s trichrome staining demonstrated slight increased collagen deposition in the myocardium of HFD-fed mice (**Figure 3C**), indicating enhanced cardiac fibrosis. These findings suggest that HFD-induced metabolic disturbances are accompanied by myocardial inflammation and fibrotic remodeling, highlighting the occurrence of early cardiovascular injury during metabolic syndrome progression.

### 3.4 HFD alters gut microbiota composition and reduces microbial community stability

To investigate whether HFD-induced metabolic abnormalities were associated with gut microbiota dysbiosis, 16S rRNA gene sequencing was performed on cecal contents collected from Ctrl and HFD mice. High-quality sequencing data were obtained, with rarefaction curves reaching saturation and Good’s coverage values approaching 1.0, indicating sufficient sequencing depth.

**Figure 4:**
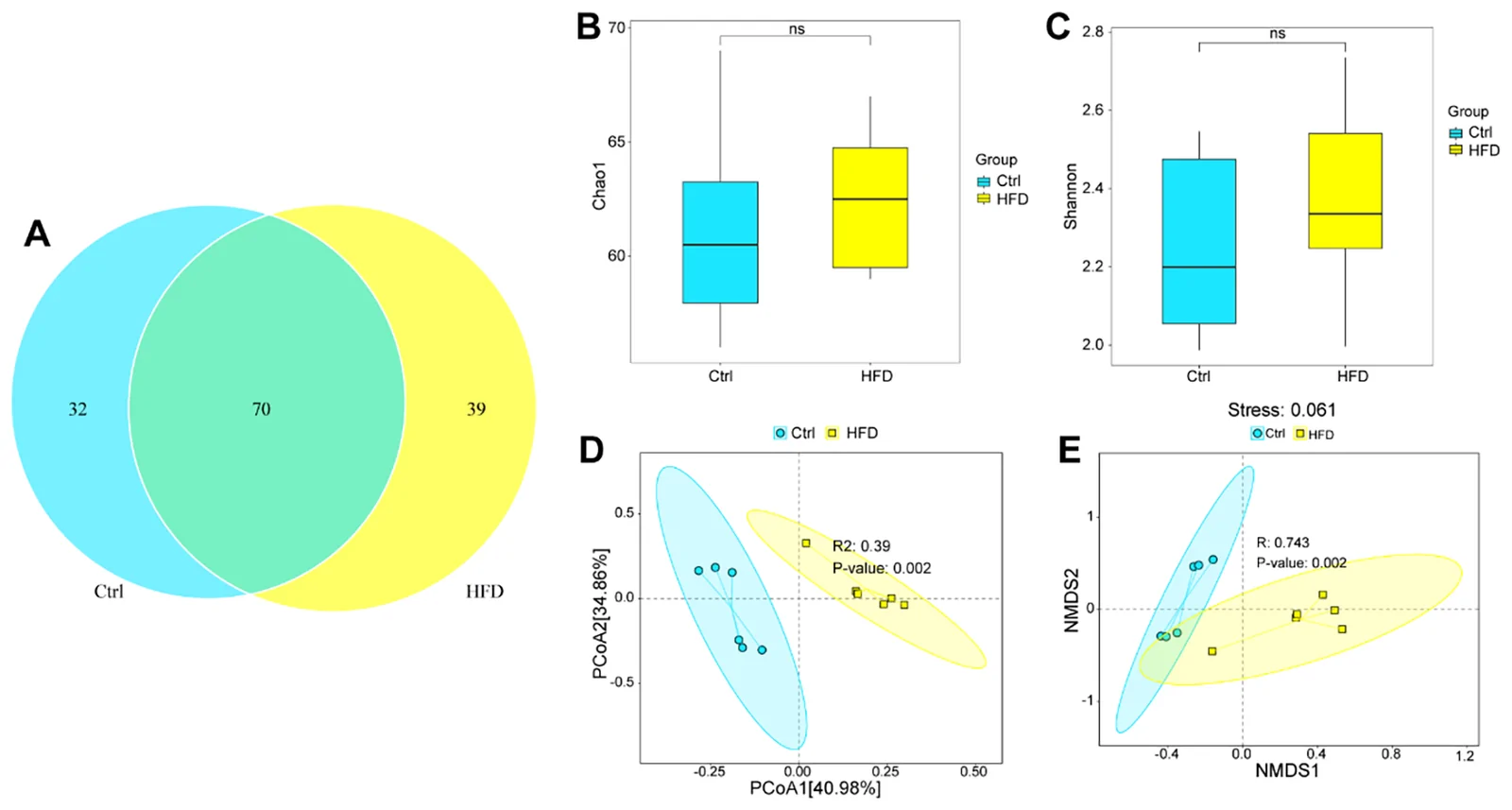
HFD alters gut microbiota composition and reduces microbial community stability. **(A)** Venn diagram showing shared and unique amplicon sequence variants (ASVs) between the Ctrl and HFD groups. **(B, C)** Alpha-diversity analysis showing the Chao1 richness index **(B)** and Shannon diversity index **(C)**. **(D, E)** Beta-diversity analyses based on principal coordinates analysis (PCoA) **(D)** and non-metric multidimensional scaling (NMDS) **(E)**.

ASV analysis identified 70 shared ASVs between the two groups, whereas 32 and 39 unique ASVs were detected in the Ctrl and HFD groups, respectively (**Figure 4A**), suggesting the presence of diet-specific microbial signatures. Taxonomic profiling revealed that individual samples contained 7–10 bacterial phyla and 56–69 genera (**Supplementary Table 1**), reflecting a moderately complex gut microbial ecosystem.

The overall taxonomic composition is presented in **Supplementary Figure 1**. At the phylum level, the gut microbiota was predominantly composed of Actinobacteriota, Bacteroidota, Deferribacterota, Desulfobacterota, Firmicutes, Proteobacteria, and Verrucomicrobiota (**Supplementary Figures 1A, G**). At the genus level, dominant taxa included Lachnospiraceae_NK4A136_group, Bacteroides, Allobaculum, [Eubacterium]_coprostanoligenes_group, Parasutterella, Escherichia-Shigella, Alloprevotella, Lachnoclostridium, Akkermansia, and Muribaculaceae (**Supplementary Figures 1E, K**).

Alpha-diversity analysis revealed no significant differences in the Chao1 or Shannon indices between groups (**Figures 4B, C**), indicating that HFD did not substantially affect overall microbial richness or diversity. In contrast, beta-diversity analyses based on principal coordinates analysis (PCoA) (**Figure 4D**) and non-metric multidimensional scaling (NMDS) (**Figure 4E**) demonstrated clear separation between Ctrl and HFD samples, indicating a pronounced shift in gut microbial community structure induced by HFD feeding. Notably, samples from the HFD group exhibited greater dispersion than those from the Ctrl group, suggesting increased inter-individual variability and reduced microbial community stability under HFD conditions.

### 3.5 LEfSe analysis identifies HFD-associated microbial biomarkers

**Figure 5:**
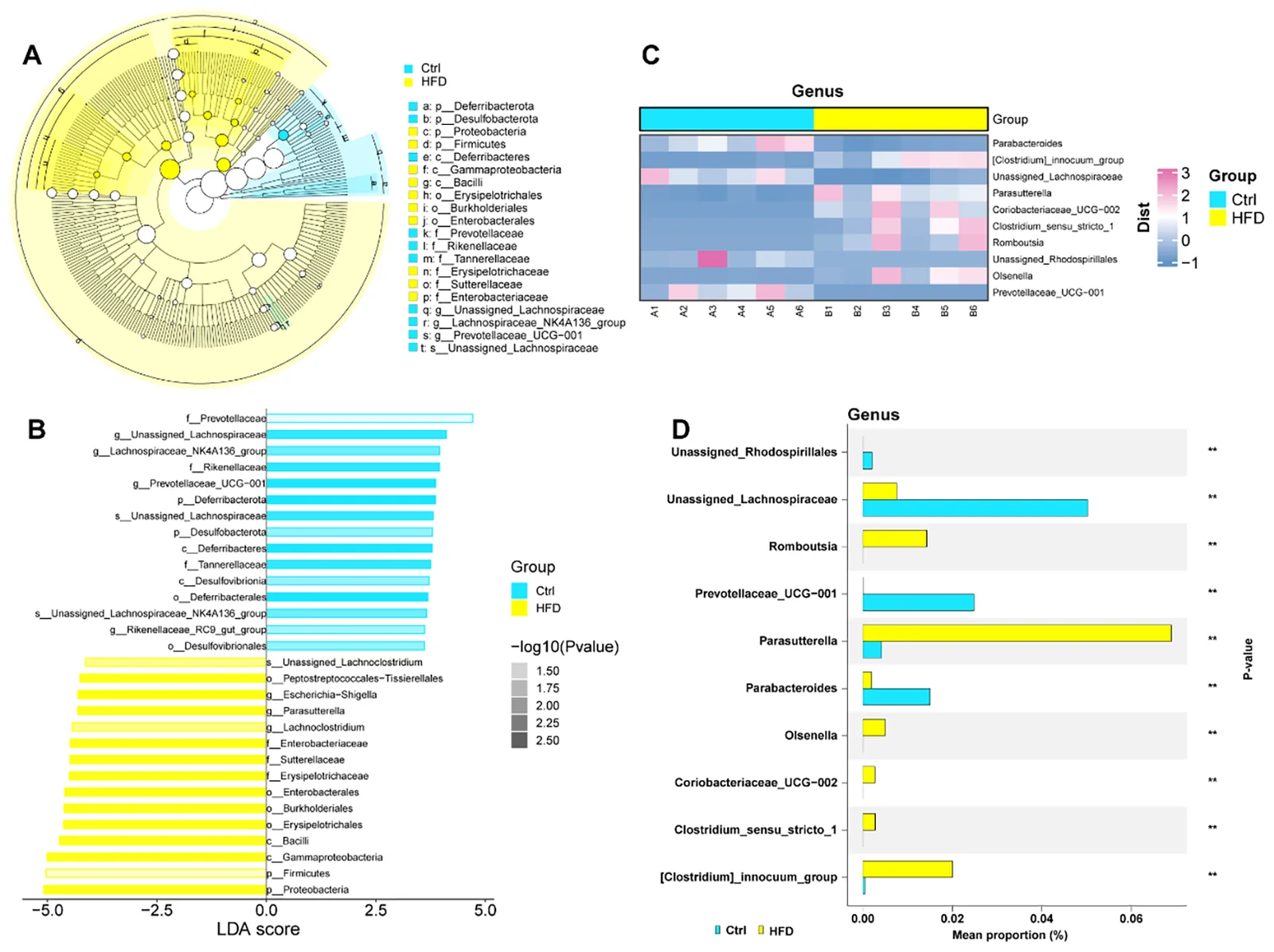
LEfSe analysis identifies HFD-associated microbial biomarkers. **(A)** Cladogram generated by linear discriminant analysis effect size (LEfSe) showing differentially enriched microbial taxa. **(B)** Histogram of LEfSe-derived taxa with significant differences between groups. **(C)** Heatmap showing the relative abundances of differential taxa at the genus level. **(D)** Differentially abundant genera identified by Wilcoxon rank-sum tests. **P < 0.01.

To identify microbial taxa that discriminated between dietary groups, linear discriminant analysis effect size (LEfSe) analysis was performed using an LDA threshold score > 3.0 (**Figures 5A, B**). A total of 15 taxa were significantly enriched in the HFD group, among which Proteobacteria showed the most pronounced increase (**Figures 5A, B**), suggesting an HFD-associated microbial shift linked to metabolic inflammation. Notably, several taxa, including *Escherichia-Shigella*, *Parasutterella*, *Alistipes*, and *Roseburia*, were consistently identified as differential taxa across multiple analytical approaches.

MetagenomeSeq analysis further revealed significant alterations in microbial composition between groups (**Supplementary Table 2**). Compared with Ctrl mice, HFD-fed mice exhibited significant enrichment of *Escherichia-Shigella, Parasutterella, Faecalibaculum, and [Clostridium]_innocuum_group*, whereas the relative abundances of *Roseburia, Alistipes, Parabacteroides, Prevotellaceae_UCG-001*, and *Rikenellaceae_RC9_gut_group* were significantly reduced (FDR-adjusted P < 0.05). These findings suggest that HFD feeding induces gut microbiota dysbiosis characterized by expansion of inflammation-associated taxa and depletion of potentially beneficial commensal bacteria.

Metastats analysis further confirmed the HFD-induced microbial alterations. Several taxa belonging to the phyla *Bacteroidota* and *Firmicutes*, including *Alistipes* and *Parabacteroides*, were significantly enriched in Ctrl mice, whereas members of *Proteobacteria* and *Erysipelotrichaceae* were significantly enriched in HFD-fed mice (FDR-adjusted P < 0.05, **Supplementary Table 3**). These results were highly consistent with those obtained from MetagenomeSeq analysis. Similar patterns of differential abundance were also observed using Wilcoxon rank-sum tests (**Figures 5C, D**) and STAMP analyses (**Supplementary Table 4**), further supporting the robustness of the observed microbial alterations.

To further explore the potential relationship between gut microbiota alterations and host phenotypes, genus-level Spearman correlation analyses were performed. Several HFD-associated genera, including *Escherichia-Shigella*, *Parasutterella*, and *Faecalibaculum*, showed positive correlations with metabolic, inflammatory, and cardiac remodeling-related parameters, whereas genera depleted after HFD feeding, including *Roseburia*, *Alistipes*, *Parabacteroides*, and *Prevotellaceae_UCG-001*, showed inverse correlations (**Supplementary Figure 2**). These findings suggest that HFD-induced microbial alterations are associated with metabolic inflammation and cardiac remodeling phenotypes, although causal relationships require further investigation.

### 3.6 Functional prediction reveals metabolic and inflammatory reprogramming of gut microbiota

**Figure 6:**
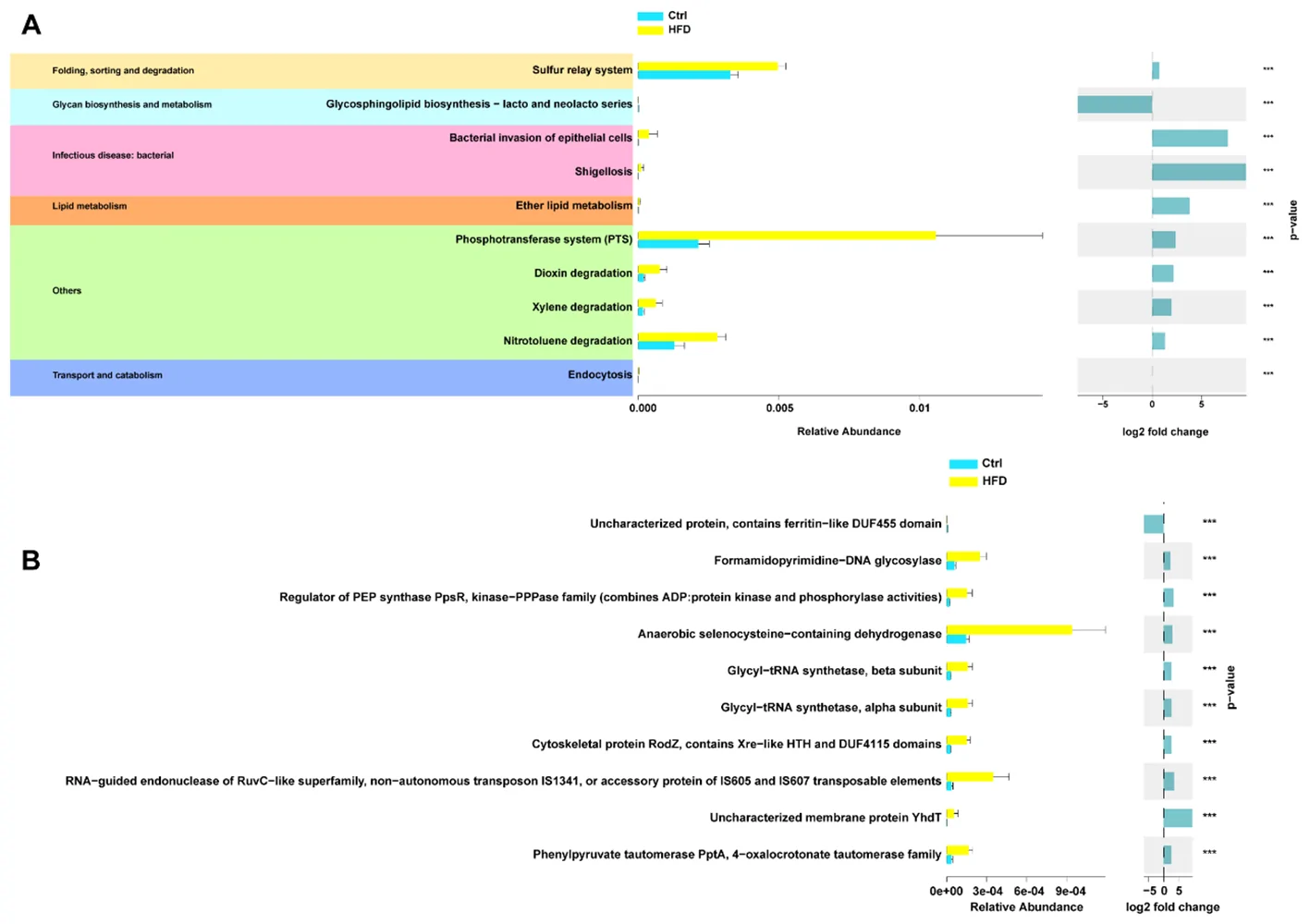
Functional prediction reveals metabolic and inflammatory reprogramming of gut microbiota. **(A)** KEGG pathway enrichment analysis showing significantly altered microbial functions between groups. **(B)** Clusters of Orthologous Groups (COG) functional enrichment analysis of the gut microbiota. ***P *<* 0.001.

To investigate the functional consequences of HFD-induced microbial alterations, KEGG and COG functional prediction analyses were performed. KEGG pathway analysis revealed significant enrichment of pathways associated with sulfur transfer systems, shigellosis, ether lipid metabolism, phosphotransferase systems (PTS), and xenobiotic degradation, including dioxin, xylene, and nitrotoluene degradation pathways, in HFD-fed mice (**Figure 6A**, **Supplementary Table 5**). These results suggest that HFD-associated microbial communities may contribute to inflammatory activation, altered lipid metabolism, and metabolic stress.

Consistently, COG functional prediction demonstrated enrichment of genes involved in DNA repair, transcriptional regulation, kinase/phosphatase activity, aminoacyl-tRNA biosynthesis, cytoskeletal regulation, RNA-guided endonuclease activity, and transposable element-associated functions (**Figure 6B**, **Supplementary Table 6**). These findings indicate enhanced microbial adaptability, stress-response capacity, and metabolic reprogramming under HFD conditions.

Taken together, functional prediction analyses suggest that HFD-induced gut microbiota dysbiosis is accompanied by profound alterations in microbial metabolic activities and inflammatory potential, which may contribute to the development of metabolic and cardiovascular dysfunction.

### 3.7 BugBase phenotype prediction reveals functional shift toward stress-adapted and potentially pathogenic microbiota

BugBase phenotype prediction analysis demonstrated that HFD feeding significantly altered microbial functional phenotypes (**Supplementary Table 7**). Compared with control mice, HFD-fed mice exhibited marked increases in facultatively anaerobic and stress-tolerant bacterial phenotypes. In addition, biofilm-forming capacity, mobile genetic element-associated traits, and potentially pathogenic phenotypes were elevated in HFD mice. Conversely, anaerobic stability was reduced and showed increased inter-individual variability in the HFD group, indicating disruption of microbial ecological homeostasis. Although Gram-negative bacteria showed only minor changes, the overall functional profile shifted toward a more stress-adapted and potentially pathogenic microbial community. Collectively, these findings suggest that HFD drives gut microbiota toward a dysbiotic functional state characterized by enhanced environmental adaptability, pathogenic potential, and reduced ecological stability.

## 4 Discussion

**Figure 7:**
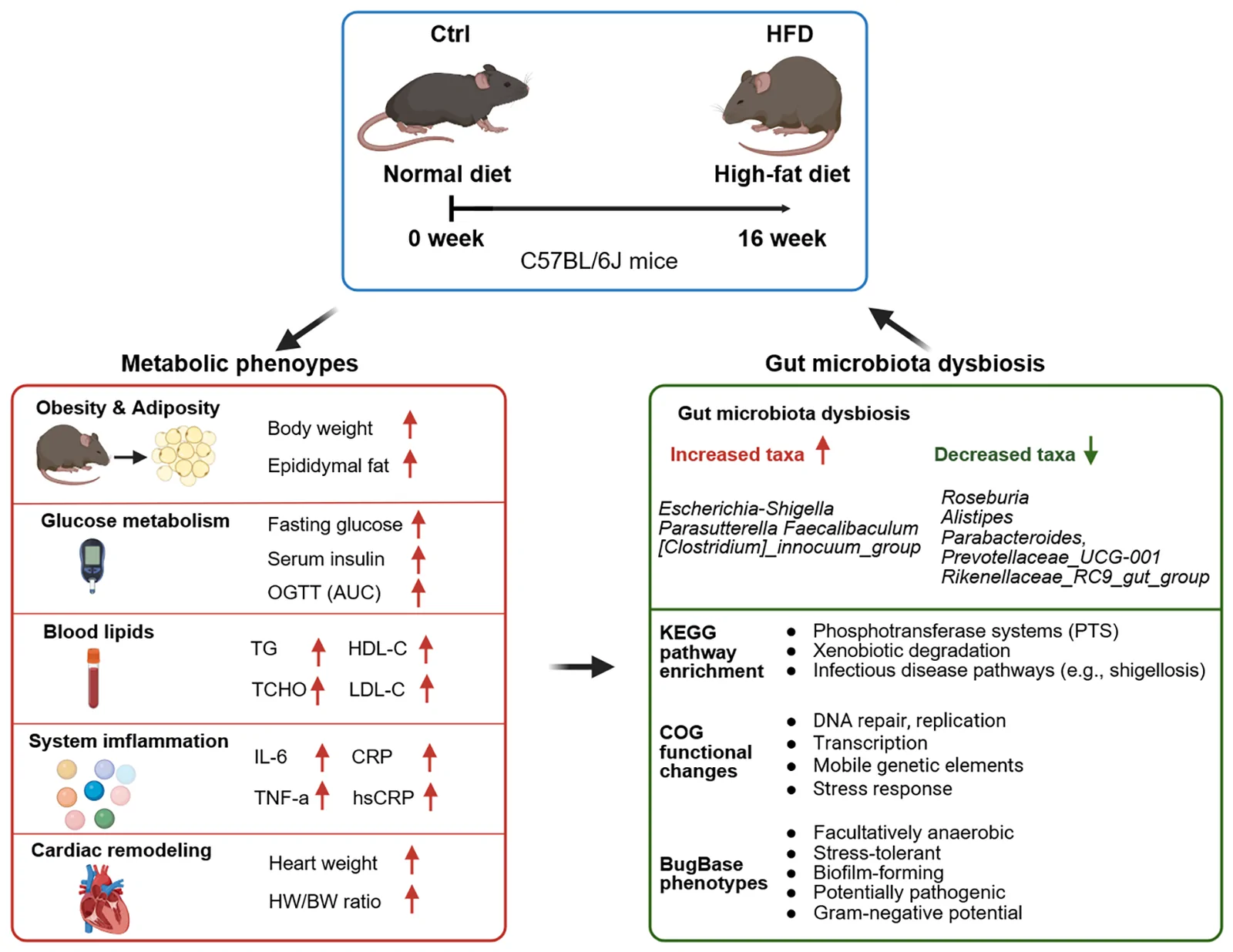
Proposed mechanism underlying HFD-induced cardiometabolic injury through the gut-heart axis. HFD induces metabolic dysfunction and gut microbiota dysbiosis, accompanied by microbial functional reprogramming characterized by increased stress-tolerant and potentially pathogenic phenotypes. These alterations promote systemic inflammation and contribute to early cardiac injury through the gut-heart axis.

In the present study, we demonstrated that long-term high-fat diet (HFD) feeding induces a metabolic syndrome (MetS)-like phenotype characterized by obesity, insulin resistance, dyslipidemia, systemic inflammation, and early cardiac remodeling in mice. Importantly, HFD exposure was accompanied by marked alterations in cecal microbiota composition and predicted functional profiles, supporting an association between gut microbiota dysbiosis and HFD-associated cardiometabolic dysfunction. However, the current study does not establish a direct causal relationship between microbial alterations and cardiac remodeling (**Figure 7**).

Firstly, HFD feeding successfully established a state of metabolic dysfunction accompanied by chronic low-grade inflammation. Consistent with previous studies, HFD-fed mice exhibited significant increases in body weight, impaired glucose tolerance, hyperinsulinemia, and dyslipidemia, all of which are hallmark features of MetS (31). These metabolic abnormalities were accompanied by elevated circulating levels of IL-6, TNF-α, CRP, and hsCRP, indicating activation of systemic inflammatory responses (35, 36). Chronic inflammation is widely recognized as a central mechanism linking obesity to cardiovascular complications. Histological analyses further revealed inflammatory cell infiltration and collagen deposition in myocardial tissue, suggesting the initiation of cardiac fibrotic remodeling. These findings support the concept that metabolic stress can induce early structural cardiac alterations even before overt cardiovascular disease becomes clinically apparent (37–39).

A major finding of the present study is that HFD exposure was associated with substantial remodeling of the cecal microbial community. Although alpha diversity remained largely unchanged, beta diversity analysis demonstrated a clear separation between control and HFD groups, indicating significant alterations in microbial community structure. Moreover, increased dispersion among HFD samples suggested reduced microbial community stability and increased ecological variability under metabolic stress conditions.

At the taxonomic level, HFD-associated microbiota alterations were characterized by enrichment of inflammation-associated taxa, including *Escherichia-Shigella*, *Parasutterella*, *Faecalibaculum*, and the *Clostridium innocuum group*, while reducing the abundance of potentially beneficial bacteria such as *Roseburia*, *Alistipes*, *Parabacteroides*, *Prevotellaceae_UCG-001*, and *Rikenellaceae_RC9_gut_group*. Among these taxa, *Escherichia-Shigella* has been linked to obesity (40), cardiovascular disease (41), chronic inflammatory disorders (42), and metabolic dysfunction (43–45), potentially through increased endotoxin production and pro-inflammatory cytokine (such as TNF-α, IL-1β, and IL-6) release (46). Similarly, *Parasutterella* has been implicated in bile acid metabolism and metabolic regulation (47–49), whereas alterations in *Faecalibaculum* and the *Clostridium innocuum group* have been associated with obesity-related inflammation (50–52).

In contrast, several bacterial taxa depleted in HFD-fed mice are closely associated with metabolic homeostasis. *Roseburia*, a well-recognized short-chain fatty acid (SCFA)-producing bacterium, has been negatively associated with obesity, insulin resistance, and dyslipidemia (53–55). Other taxa, including *Parabacteroides*, *Alistipes*, *Prevotellaceae_UCG-001*, and *Rikenellaceae_RC9_gut_group*, have been implicated in SCFA production, bile acid metabolism, intestinal barrier maintenance, and regulation of host metabolic balance (40, 48, 56–59). Collectively, these alterations indicate a transition from a transition toward a less metabolically favorable microbial ecosystem under HFD exposure (**Figure 7**).

Beyond compositional changes, functional prediction analyses revealed substantial remodeling of microbial functional potential following HFD exposure. KEGG and COG analyses demonstrated alterations in pathways related to phosphotransferase systems, xenobiotic metabolism, stress responses, transcriptional regulation, DNA repair, and mobile genetic elements. These findings indicate that HFD-associated microbial communities may undergo functional adaptation in response to dietary metabolic stress. However, because these analyses were based on 16S rRNA sequencing-based prediction, further metagenomic and metabolomic studies are required to validate these functional changes.

BugBase phenotype prediction further suggested increased facultatively anaerobic, stress-tolerant, biofilm-forming, and potentially pathogenic bacterial phenotypes in HFD-fed mice. These findings indicate a shift toward a stress-adapted microbial ecological state. However, these predicted phenotypes require experimental validation.

Previous studies have demonstrated that microbial-derived metabolites may represent important mediators linking gut microbiota alterations with host metabolic and cardiovascular abnormalities. These experimentally supported mechanisms provide a biological framework for interpreting the microbial changes observed in our study (40, 60–62). It has been demonstrated that SCFAs regulate immune homeostasis, intestinal barrier integrity, and inflammatory responses through GPR41/43 signaling and epigenetic regulation (63–65). Therefore, depletion of SCFA-producing bacteria under HFD conditions may contribute to impaired microbial metabolic signaling and systemic inflammation (66, 67). Similarly, microbiota-dependent bile acid transformation regulates FXR and TGR5 pathways (68–70), which play important roles in metabolic regulation and anti-inflammatory responses (49, 71, 72).

Emerging evidence also indicates a bidirectional relationship between metabolic syndrome and infections caused by multidrug-resistant (MDR) pathogens. MetS-associated chronic inflammation, impaired immune responses, visceral adiposity, and gut microbiota imbalance may influence susceptibility to microbial colonization and infection (73). Conversely, severe infections, particularly those caused by multidrug-resistant pathogens, may induce systemic inflammatory activation and metabolic disturbances, further disrupting host-microbiota interactions (74–76). Incorporating this perspective highlights the broader clinical relevance of microbiota-mediated immune-metabolic regulation.

In addition to microbial metabolites, bacterial-derived toxins and microbial components may represent another potential mechanism linking gut dysbiosis with cardiovascular dysfunction (77, 78). Certain bacterial toxins can influence host cell membrane integrity, ion homeostasis, and inflammatory signaling, thereby affecting cardiac function (78, 79). Nevertheless, the direct contribution of specific microbial toxins to HFD-induced cardiac remodeling remains unclear and requires further investigation.

Together, our findings support a potential association between HFD-induced gut microbiota dysbiosis, systemic inflammation, and early cardiac remodeling. Previous experimental studies have demonstrated causal roles for microbial metabolites, intestinal barrier dysfunction, and inflammatory signaling pathways in cardiovascular regulation (80, 81). However, our current findings derived from 16S rRNA sequencing and phenotypic correlation analyses remain associative. Therefore, whether specific microbial taxa or microbial-derived metabolites directly mediate HFD-induced cardiac remodeling requires further validation using causal experimental approaches.

Given the important role of gut microbiota in regulating metabolic inflammation, microbial metabolites, and host immune responses, microbiota-targeted interventions, including dietary modification (82, 83), probiotics/prebiotics (84, 85), fecal microbiota transplantation (86), and metabolite-based therapies (87, 88), represent potential strategies for obesity-related cardiovascular disease prevention. Future studies integrating microbiota manipulation models and multi-omics approaches will be necessary to identify specific microbial targets and develop effective microbiota-based therapies.

Several limitations should be acknowledged. First, the sample size was relatively limited. Second, functional predictions derived from 16S rRNA sequencing provide indirect evidence of microbial activity and require validation using metagenomic, metatranscriptomic, and metabolomic approaches. Third, although associations between gut microbiota alterations and cardiac remodeling were observed, causal relationships were not experimentally established. Future microbiota manipulation studies will be necessary to validate the proposed gut–heart axis mechanisms.

## 5 Conclusion

In summary, our findings suggest that gut microbiota alterations are associated with metabolic inflammation and early cardiac remodeling during HFD exposure, highlighting the gut microbiota as a potential contributor to obesity-related cardiometabolic dysfunction. Further mechanistic studies are required to establish causal links within the gut-heart axis.

## Data Availability

The raw sequencing data supporting the findings of this study have been deposited in the Genome Sequence Archive (GSA) at the National Genomics Data Center (NGDC) under accession number CRA046833 and are accessible at https://ngdc.cncb.ac.cn/gsa/s/2742BlXw.
