# Supplementary material for: High-fat diet-induced gut microbiota functional reprogramming is associated with metabolic inflammation and cardiac remodeling through the gut-heart axis in mice
Source: Front Immunol. 2026 Aug 25;17:1912486. doi: 10.3389/fimmu.2026.1912486 (PMC13549916; doi:10.3389/fimmu.2026.1912486)
Supplement: Supplementary file 1 [file DataSheet2.docx]

**Figure S1. Overall taxonomic composition of the gut microbiota in the Ctrl and HFD groups.**

(A-F) Relative abundances of gut microbiota at the phylum (A), class (B), order (C), family (D), genus (E), and species (F) levels in the Ctrl and HFD groups.

**Figure S2. Spearman correlation analysis between altered gut microbiota and host phenotypic parameters in HFD-fed mice.**

(A) Correlation heatmap showing the associations between selected gut microbial genera and metabolic phenotypes, including triglycerides (TG), total cholesterol (TC), high-density lipoprotein cholesterol (HDL), low-density lipoprotein cholesterol (LDL), glucose (GLU), insulin (INS), and body weight at 16 weeks (BW_16w).

(B) Correlation heatmap showing the associations between selected gut microbial genera and inflammatory/cardiac phenotypes, including interleukin-6 (IL-6), tumor necrosis factor-α (TNF-α), C-reactive protein (CRP), high-sensitivity C-reactive protein (hsCRP), and heart weight/body weight ratio (HW/BW).

Values in each cell represent Spearman’s correlation coefficients (ρ).
